# High-Amylose Corn Starch Regulated Gut Microbiota and Serum Bile Acids in High-Fat Diet-Induced Obese Mice

**DOI:** 10.3390/ijms23115905

**Published:** 2022-05-25

**Authors:** Jiamiao Hu, Peiying Zheng, Jinhui Qiu, Qingyan Chen, Shaoxiao Zeng, Yi Zhang, Shaoling Lin, Baodong Zheng

**Affiliations:** 1 Engineering Research Centre of Fujian-Taiwan Special Marine Food Processing and Nutrition, Ministry of Education, Fuzhou 350002, China; jiamiao.hu@fafu.edu.cn (J.H.); zhengpy19990520@163.com (P.Z.); cqyqingyan@outlook.com (Q.C.); zsxfst@163.com (S.Z.); zyifst@163.com (Y.Z.); 2 Key Laboratory of Marine Biotechnology of Fujian Province, Institute of Oceanology, Fujian Agriculture and Forestry University, Fuzhou 350002, China; qjh6511213@163.com; 3 College of Food Science, Fujian Agriculture and Forestry University, Fuzhou 350002, China

**Keywords:** high amylose corn starch, nutritive obesity, gut microbiota, bile acids

## Abstract

**Simple Summary:**

High-amylose corn starch, as a kind of resistant starch, could profoundly regulate the gut microbiota and exert anti-obesity properties. Since the gut microbiota was found to improve metabolic health by altering circulating bile acids, therefore, here we investigated the association between the gut microbiota and serum bile acids in high fat diet induced obese mice fed with high-amylose corn starch. We found high-amylose corn starch could modulate the gut microbiota composition and partially restore the alternations in circulating bile acid profiles in obese mice. These influences on gut microbiota and circulating bile acids could be the underlying mechanisms of anti-obesity activity of high-amylose corn starch.

**Abstract:**

High-amylose corn starch is well known for its anti-obesity activity, which is mainly based on the regulatory effects on gut microbiota. Recently, the gut microbiota has been reported to improve metabolic health by altering circulating bile acids. Therefore, in this study, the influence of high-amylose corn starch (HACS) on intestinal microbiota composition and serum bile acids was explored in mice fed with a high fat diet (HFD). The results demonstrated HACS treatment reduced HFD-induced body weight gain, hepatic lipid accumulation, and adipocyte hypertrophy as well as improved blood lipid profiles. Moreover, HACS also greatly impacted the gut microbiota with increased *Firmicutes* and decreased *Bacteroidetes* relative abundance being observed. Furthermore, compared to ND-fed mice, the mice with HFD feeding exhibited more obvious changes in serum bile acids profiles than the HFD-fed mice with the HACS intervention, showing HACS might restore HFD-induced alterations to bile acid composition in blood. In summary, our results suggested that the underlying mechanisms of anti-obesity activity of HACS may involve its regulatory effects on gut microbiota and circulating bile acids.

## 1. Introduction

Obesity has reached global epidemic proportions [1] and imposes a large economic burden on society by increasing disability, rising health care costs and reducing life expectancy [2]. The prevalence of obesity is a result of various factors, including the popularity of calorie-dense foods, sedentary lifestyles [3], and even mental stress [4]. Obesity is hazardous to health and can increase the risk of cardiovascular diseases [5,6], diabetes [7], fatty liver [8], kidney diseases [9], etc.

High-amylose corn starch (HACS) is well known for its anti-obesity effect. For instance, Aziz et al. reported that rats fed with HACS showed significantly less body weight gain and increased insulin sensitivity [10]. Similarly, it was found that HACS treatment could reduce abdominal fat in rodents [11,12]. Moreover, resistant starch could also exert beneficial metabolic effects via reshaping the composition, diversity, and abundance of gut microbiota [13]. For example, it has been reported that HACS could act as a prebiotic and promoted the growth of *Bifidobacterium* spp. [14].

In the last decades, more details about how gut microbiota influence host metabolism have been revealed [15]. Improving the gut microbiota is regarded as a promising strategy to combat obesity [16,17]. For example, SCFAs, the fermentation end-products of HACS by gut microbiota, were reported to prevent obesity via multiple mechanisms, including maintaining the intestinal barrier [18], stimulating GLP-1, PYY and leptin secretion [19], increasing the production of anti-inflammatory mediators [20], reducing hepatic secretion of proinflammatory cytokines [21], slowing fat and cholesterol production [22,23], and inhibiting appetite [24]. The gut microbiota also plays crucial roles in bile acid metabolism. It is widely acknowledged that the primary bile acids synthesized in the liver can be further metabolized into secondary bile acids by colonic bacteria [25]. A recent study also showed that a lack of gut microbes in germ-free mice could even influence the expression of bile acids synthesis and transport-related genes in the liver [26]. Therefore, gut microbiota plays crucial roles in the regulation of the bile acid pool size and composition [27].

Notably, recent studies showed that changes in the gut microbiota could also influence the circulating levels of bile acids and play important roles in metabolic improvement [28]. For instance, blueberry extract was found to decrease plasma bile acids size and serum conjugated bile acids content through regulating the gut microbiota [29]. Similarly, Li et al. also reported polybrominated diphenyl ethers altered the gut microbiome and decreased serum conjugated bile acids in male C57BL/6 mice [26]. Moreover, the modulation of circulating bile acid (BA) levels was also proven to be associated with body energy homeostasis [30,31]. Indeed, bile acid receptors (e.g., TGR5 & FXR) have been found to be widely expressed in various tissues (including adipose tissue [32], liver [33], kidney [34], intestine [35], immune cells [36], cardiovascular system, brain [37], adrenal glands [38], thyroid gland, etc.) and exert important roles in lipid, glucose and energy homeostasis. For instance, vertical sleeve gastrectomy (VSG) was found to increase circulating BA concentrations and improve glucose homeostasis via activating TGR5 [39]. TGR5 activation also promotes adipose tissue browning and energy metabolism [40]. Therefore, it is believed that bile acid levels in blood crucially affect energy homeostasis.

Considering the considerable impact of high-amylose corn starch on the gut microbiota, it is reasonable to hypothesize that HACS can also influence circulating bile acid levels to exert, at least partially, its anti-obesity effect. To test this hypothesis in the current study, we determined the beneficial effects of HACS on body weight management in high-fat diet-fed mice and explored its influence on the intestinal bacterial community with changes in serum bile acids. The obtained results may provide new evidence to understand the mechanisms of anti-obesity action of HACS and highlight its therapeutic potential.

## 2. Results

### 2.1. The Effects of HACS on Body Weight Gain in HFD-Fed Mice

**Figure 1:**
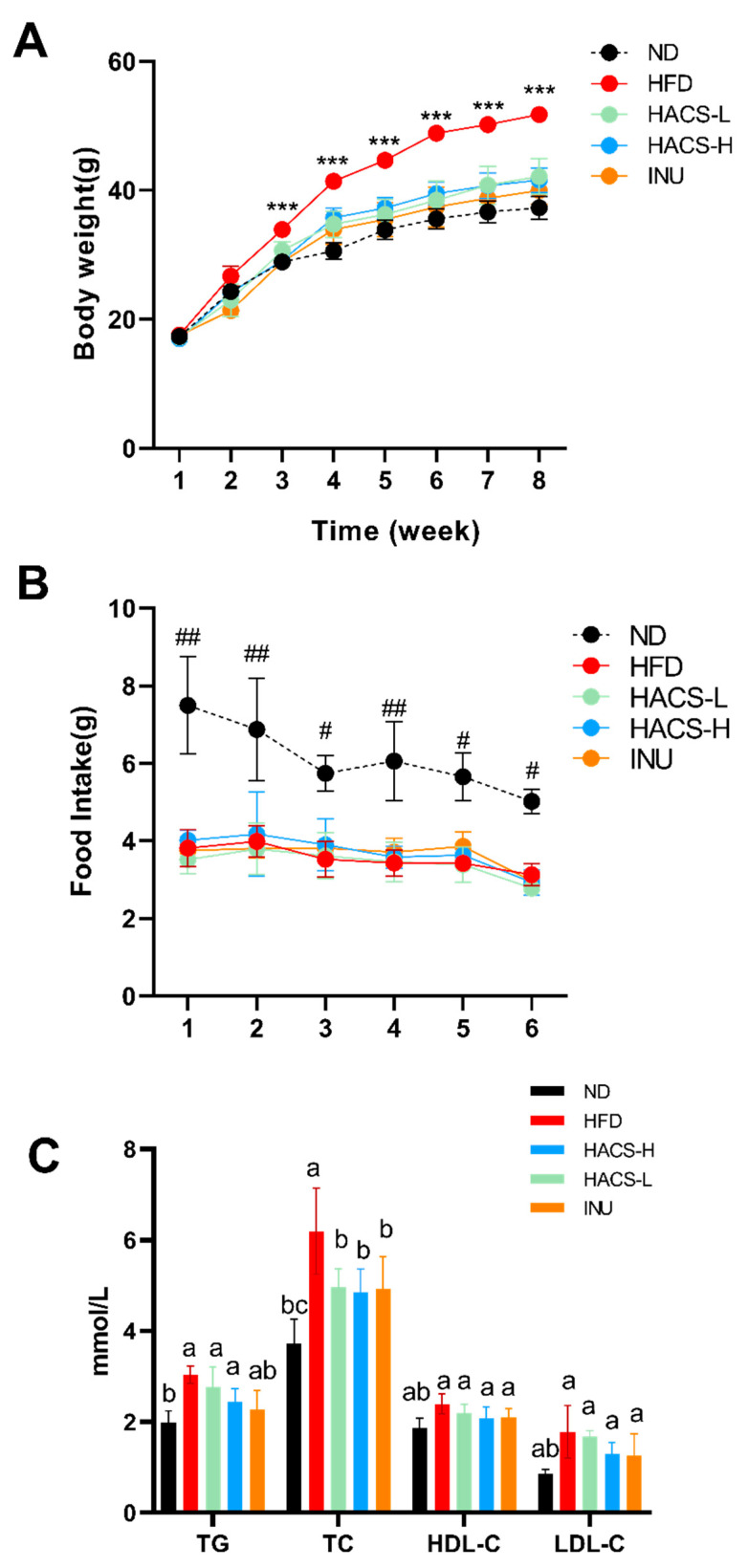
Effects of HACS treatment on the body weight (**A**), food intake (**B**), and blood lipid levels (**C**) of ICR mice. ICR mice were fed each diet for 7 weeks: ND, normal diet; HFD, high fat diet; HACS-L, high fat diet + 0.25 g/kg HACS; HACS-H, high fat diet + 0.5 g/kg HACS; INU, high fat diet + 0.5 g/kg inulin. Data were expressed as the mean ± SD (*n* = 5 per group). *** *p* < 0.001 vs. ND, INU, HACS-L and HACS-H (**A**); ^#^ *p* < 0.05 and ^##^ *p* < 0.01 vs. HFD (**B**); different lowercase letters represent significant differences (*p* < 0.05), and the same lowercase letters represent no significant differences (*p* ≥ 0.05) (**C**).

As shown in Figure 1A, no significant difference in body weight was observed among the groups before intervention. Consistent with previous studies, HFD feeding for 8 weeks resulted in a significant increase in mice body weight (red curve, *p* < 0.01). In addition, intervention with inulin or HACS during the HFD feeding effectively retarded the body weight gain. Notably, the food intake of mice with HACS treatments was similar to that of HFD-fed mice (Figure 1B), indicating this the body weight-lowering effects of HACS may not result from appetite inhibition.

### 2.2. The Effects of HACS on Blood Lipid Levels in HFD-Fed Mice

We next determined the effects of HACS on blood lipid profile. As shown in Figure 1C, HFD-fed mice exhibited significantly higher circulating levels of TG, TC, and LDL-C levels compared to ND groups. Notably, the HACS treatment could significantly lower the circulating TC level (*p* = 0.0269), though decreases in TG, HDL-C, and LDL-C levels did not reach statistical significance. The positive control inulin also exerted similar effects on circulating lipid profile as HACS treatment.

### 2.3. The Effects of HACS on Adipose Tissues in HFD-Fed Mice

**Figure 2:**
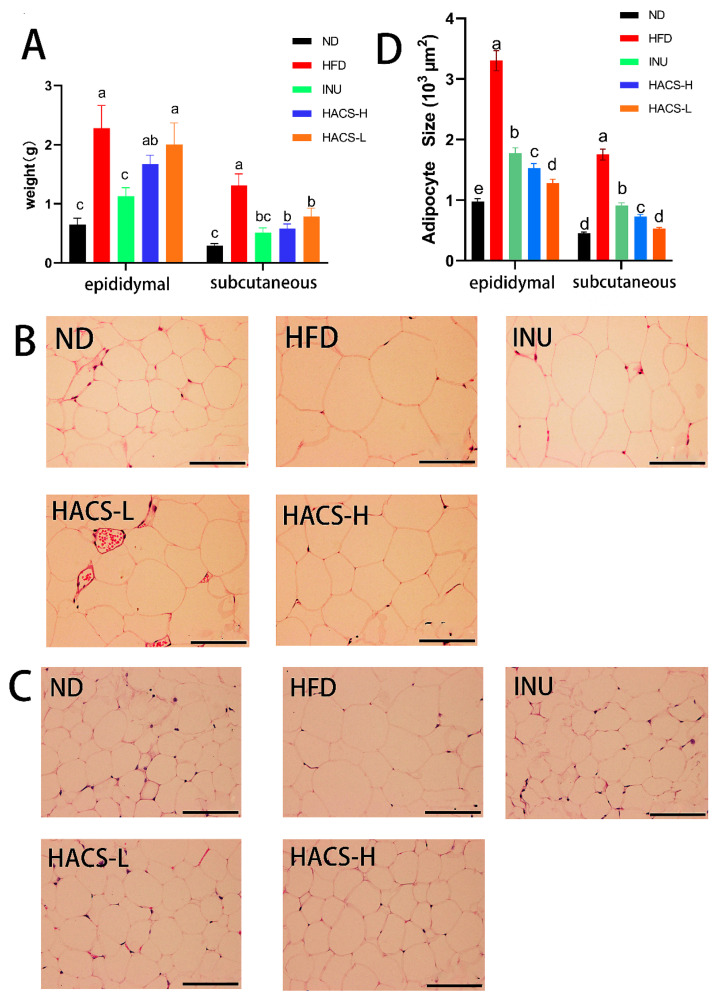
Effects of HACS treatment on adipose tissue. (**A**) the weight of epididymal and subcutaneous adipose tissues; (**B**) the morphology of epididymal adipocytes; (**C**) the morphology of subcutaneous adipocytes; and (**D**) the average adipocyte size. ND, normal diet; HFD, high fat diet; HACS-L, high fat diet + 0.25 g/kg HACS; HACS-H, high fat diet + 0.5 g/kg HACS; INU, high fat diet + 0.5 g/kg inulin. Data were expressed as the mean ± SD (*n* = 5 per group). Different lowercase letters represent significant differences (*p* < 0.05), and the same lowercase letters represent no significant differences (*p* ≥ 0.05). The scale bars represent 100 μm.

Next, the effects of HACS on adipose tissues were explored. Consistent with its body weight lowering effect, HACS treatment also significantly decreased the weight of subcutaneous adipose tissue, though only subtle changes in the weight of epididymal adipose tissue were observed (Figure 2A). Furthermore, since obesity is often characterized by adipocyte hypertrophy [41], we also evaluated the average cell size of epididymal and subcutaneous adipose tissue depots. As shown in Figure 2, enlarged adipocytes were found in both epididymal and subcutaneous adipose tissues in the HFD group when compared with the ND group, indicating that the HFD led to adipocyte hypertrophy in adipose tissue in mice. Meanwhile, the obtained results also demonstrated HACS and inulin showed similar inhibitory effects on adipocyte hypertrophy. The quantitative analysis also showed HACS and inulin significantly decreased the average size of adipocytes in subcutaneous and epididymal adipose tissues in mice with HFD feeding (*p* < 0.05) (Figure 2D).

### 2.4. The Effects of HACS on Hepatic Steatosis in HFD-Fed Mice

**Figure 3:**
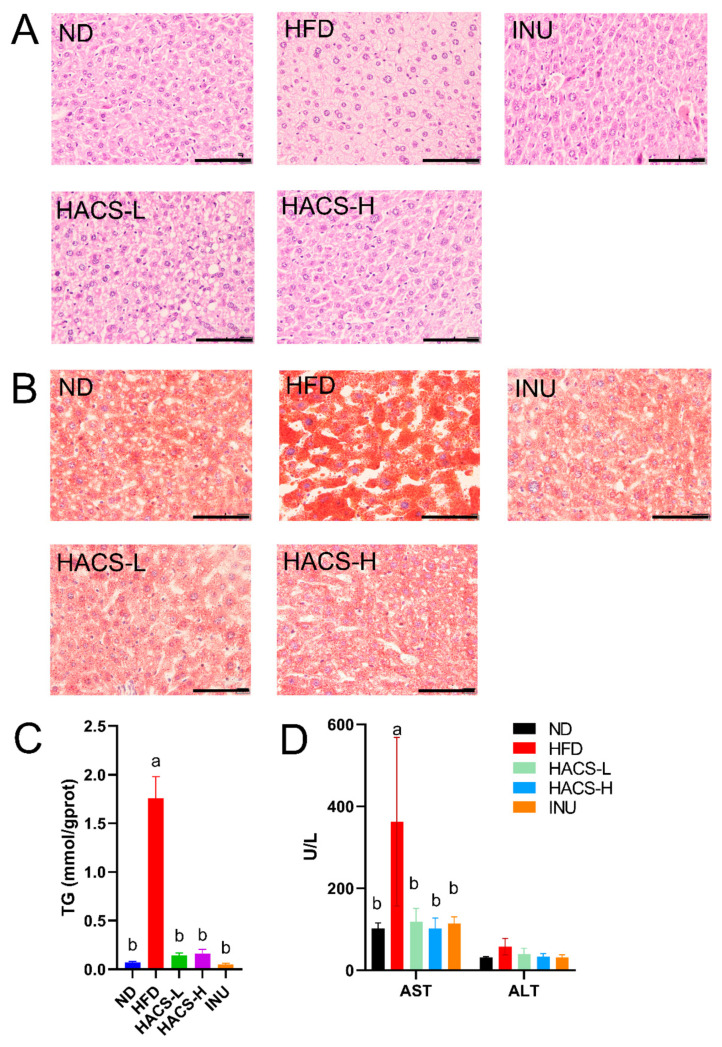
Effects of HACS treatment on liver tissue. HE staining (**A**), Oil Red O staining (**B**), the content of TG (**C**), and the levels of ALT and AST (**D**) of the liver. ND, normal diet; HFD, high fat diet; HACS-L, high fat diet + 0.25 g/kg HACS; HACS-H, high fat diet + 0.5 g/kg HACS; and INU, high fat diet + 0.5 g/kg inulin. Data were expressed as the mean ± SD (*n* = 5 per group). Different lowercase letters represent significant differences (*p* < 0.05), and the same lowercase letters represent no significant differences (*p* ≥ 0.05). The scale bars represent 100 μm.

HFD-induced obesity is also closely associated with hepatic steatosis [42] and elevated hepatic AST and ALT levels [43]. As shown in Figure 3A, the HE staining revealed that HFD feeding resulted in obvious histopathological changes in the liver, while HACS supplementation as well as the positive control (inulin) were found to alleviate liver vacuoles induced by HFD. The Oil Red O staining also showed that HFD-induced hepatic lipid accumulation could be ameliorated by HACS (Figure 3B). Meanwhile, quantitative analysis of hepatic TG levels revealed the HFD feeding significantly elevated the TG contents in the liver when compared to that of ND-fed mice, while supplementation of HACS and inulin lowered HFD-induced hepatic TG accumulation (Figure 3C). Furthermore, HACS treatment also significantly reduced HFD-induced elevation in hepatic AST activities (*p* < 0.05); while similar lowering effects on ALT activities were also observed (though not reaching statistical significance), indicating that HACS could effectively protect hepatocytes damaged by HFD (Figure 3D). These results suggested that HACS could ameliorated the hyperlipidemia caused by HFD feeding.

### 2.5. The Influences of HACS on Gut Microbiota Composition

**Figure 4:**
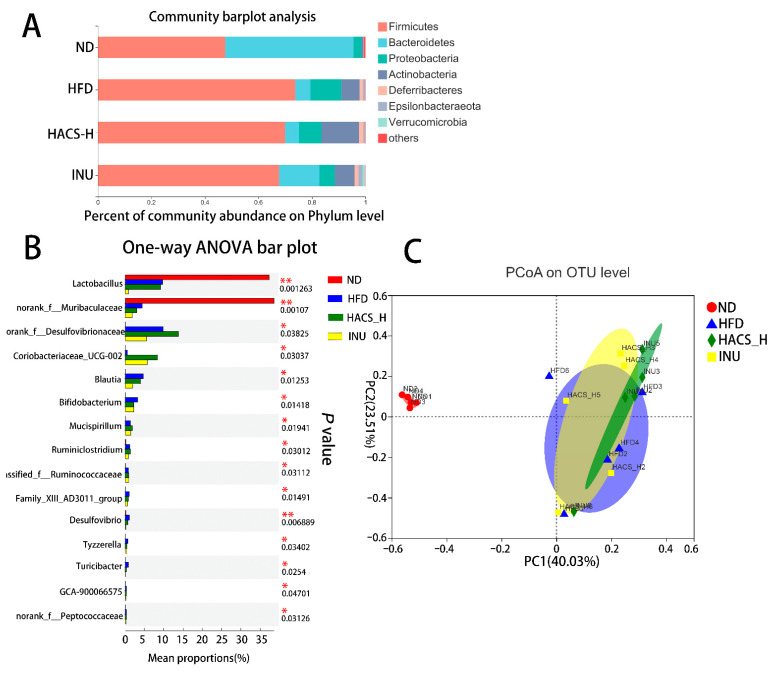
Effects of HACS treatment on gut microbiota composition. (**A**) Community abundance of gut microbiota on phylum level; (**B**) one-way ANOVA analysis of gut microbiota composition on genus level, and (**C**) PCoA analysis of the bacterial community on OTU level. ND, normal diet; HFD, high fat diet; HACS-L, high fat diet + 0.25 g/kg HACS; HACS-H, high fat diet + 0.5 g/kg HACS; INU, high fat diet + 0.5 g/kg inulin (*n* = 5 per group).

The effects of HACS on intestinal microflora of obese mice were studied next. At the phylum level, as expected, HFD induced an obvious increase in *Firmicutes* and decrease in *Bacteroidetes*, which has been identified as characteristic dysbiosis usually observed with obesity. Meanwhile, the results also demonstrated that intragastrical administration of HACS led to a decrease in *Firmicutes* abundance; while inulin demonstrated a similar effect with decreased *Firmicutes* abundance and increased *Bacteroidetes* abundance being observed (Figure 4A).

At the genus level, the ANOVA analysis also revealed the dietary intervention significantly altered the gut microbiota (*p* < 0.05). For instance, the high-fat diet intervention resulted in significantly lower relative abundances of *Lactobacillus*, *norank_f__Muribaculaceae* and increased the relative abundances of *norank_f__Desulfovibrionaceae*. Meanwhile, consumption of HACS reversed the HFD-induced changes in bacteria: *Blautia*, *Bifidobacterium*, *Family_XIII_AD3011_group*, *Desulfovibrio*, *Tyzzerella*, *Turicibacter*. The inulin intervention also exerted a similar effect as the HACS treatment (Figure 4B).

The principal component analysis (PCoA) diagram also supported this finding that there existed a distinct separation between the ND group and the HFD group, while the HACS and inulin groups shared a high similarity (Figure 4C).

### 2.6. The Influences of HACS on Serum Bile Acid Levels

**Figure 5:**
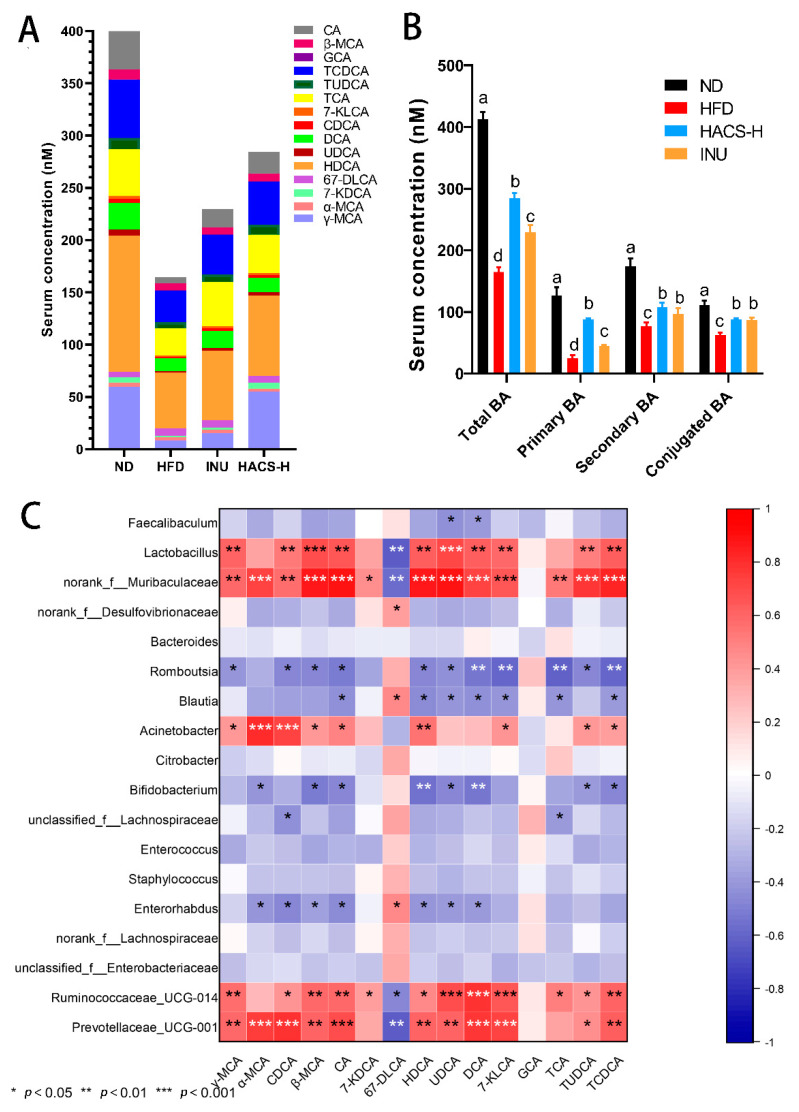
Effects of HACS treatment on serum bile acids. (**A**) Concentration of 15 individual bile acids in serum; (**B**) concentration of primary, secondary, and conjugated bile acids in serum, and (**C**) Spearman correlation between bacterial composition and bile acids. ND, normal diet; HFD, high fat diet; HACS-L, high fat diet + 0.25 g/kg HACS; HACS-H, high fat diet + 0.5 g/kg HACS; and INU, high fat diet + 0.5 g/kg inulin. CA: cholic acid; β-MCA: β-mouse cholic acid; GCA: glycocholic acid; TCDCA: sodium taurochenodeoxycholic acid; TUDCA: tauroursodeoxycholic acid; TCA: taurocholic acid; 7-KLCA: 7-ketocornerstone cholic acid; CDCA: chenodeoxycholic acid; DCA: deoxycholic acid; UDCA: ursodeoxycholic acid; HDCA: hyodeoxycholic acid; 67-DLCA: 6, 7-diketolithocholic acid; 7-KDCA: 7-ketodeoxycholic acid; α-MCA: α-mouse cholic acid; and γ-MCA: gamma-mouse cholic acid. Data were expressed as the mean ± SD (*n* = 5 per group). Different lowercase letters represent significant differences (* *p* < 0.05), and the same lowercase letters represent no significant differences (* *p* ≥ 0.05) (**B**); red represents a positive correlation, and blue represents a negative correlation. * *p* < 0.05, ** *p* < 0.01, *** *p* < 0.001 (**C**).

The bile acids composition in the serum of mice following different treatments is shown in Figure 5 and Table 1. In general, circulating bile acids (including conjugated bile acids, secondary bile acids, and primary bile acids) decreased significantly upon HFD-feeding (*p* < 0.01), while HACS administration slightly but significantly elevated (*p* < 0.05) the circulating levels of bile acids in obese mice. Moreover, as shown in Table 1, the circulating levels of nearly all tested bile acids (except glycocholic acid (GCA) and 6, 7-diketolithocholic acid (67-DLCA)) decreased significantly in the HFD group (*p* < 0.01). Notably, the HACS treatment almost restored the circulating gamma-mouse cholic acid (γ-MCA), 7-ketodeoxycholic acid (7-KDCA), and GCA to the levels in the ND group, while hyodeoxycholic acid (HDCA), 7-ketocornerstone cholic acid (7-KLCA), taurocholic acid (TCA), tauroursodeoxycholic acid (TUDCA), sodium taurochenodeoxycholic acid (TCDCA), β-mouse cholic acid (β-MCA), and cholic acid (CA) levels were also significantly increased by HACS treatment. Similar effects were also observed in the positive control group treated with inulin (Table 1).

Next, the correlation between bile acid levels in blood and gut microbiota composition were further analyzed. As shown in Figure 5C, the abundance of *Prevotellaceae_UCG-001, Ruminococcaceae_UCG-014*, *Acinetobacter*, *Lactobacillus* and *norank_f__Muribaculaceae* were significantly positively correlated with most bile acids (*p* < 0.05); while *Enterorhabdus, Blautia*, *Bifidobacterium* and *Romboutsia* were significantly negatively correlation with most bile acids (*p* < 0.05). Notably, the correlation between bile acid 67-DLCA and the gut microbiota showed an opposite trend when compared to other bile acids.

## 3. Discussion

In this study, we determined the anti-obesity effects of HACS in HFD-induced obese mice and explored its possible underlying mechanisms. Particularly, the regulatory effects of HACS on gut microbes and circulating bile acids were investigated.

As expected [44], upon 7 weeks of HFD feeding, mice developed obesity-like changes, including rapid body weight gain, dyslipidemia, adipocyte hypertrophy, and hepatic steatosis. Furthermore, consistent with previous findings [45,46], the HACS intervention clearly showed the anti-obese effects evidenced by lower body weight, improved blood lipid profiles, reduced size of adipocytes in adipose tissue, and relieved steatohepatitis. Indeed, these observations were also supported by evidence from human trials. For instance, overweight and obese men received 15 g/d and 30 g/d of high-amylose maize type 2 resistant starch that improved insulin sensitivity [47].

Notably, our obtained results showed that HACS treatment did not significantly affect food intake. This observation is consistent with previous findings that feeding resistant starch could reduce body fat in rodent models without obvious effects on food intake [48]. Indeed, a number of mechanisms have been proposed to explain the anti-obesity effects of resistant starch. For example, resistant starch was reported to enhance energy expenditure via increasing the physical activity [49] and thermic effect of food in mice [50]. These may contribute, at least partially, to the beneficial metabolic effects of HACS.

Moreover, the impact of resistant starch on gut microbiota also received considerable attention. Here, our obtained results also confirmed that HACS and inulin administration profoundly affected the gut microbiota. Particularly, HACS treatment reduced the HFD-induced increase in a variety of bacterial species positively related to obesity (e.g., *Blautia*, *Desulfovibrio*, *Tyzzerella*, and *Turicibacter*). A number of similar observations were also reported in previous literature. For instance, a multiphase dietetic protocol incorporating an improved ketogenic diet (MDP-i-KD) enhanced weight loss and altered the gut microbes in obese subjects with the relative abundance of *Blautia* being significantly decreased [51]. Here, in addition, resveratrol, a polyphenol well-known for its anti-obesity and antidiabetic health effects, was also found to decrease the relative abundance of family *Lachnospiraceae* [52] and *Desulfovibrio* [53]. Meanwhile, theabrownin, which could relieve diabetes and obesity, was also reported to reduce the growth of *Tyzzerella* while consuming a high sugar diet [54]. *Turicibacter* and *Romboutsia* were positively correlated with indicators of obesity-like TG, TC, and insulin. Similar to HACS treatment in the current study, the abundance of these two bacteria was reduced upon the intake of banana resistant starch [55]. In addition, previous studies also showed that HACS increased the abundance of bacteroides [56] that could reduce the hepatic fat accumulation and the secretion of pro-inflammatory cytokines [57], which was also observed in the current study.

Furthermore, HACS also profoundly affected the serum bile acids in obese mice. Overall, HACS treatment upregulated the circulating levels of conjugated, primary, and secondary bile acids in mice with HFD feeding. Among 15 individual bile acids tested in the current study, HACS treatment significantly increased the levels of γ-MCA, 7-KDCA, HDCA, 7-KLCA, TCA, TUDCA, TCDCA, β-MCA, and CA and decreased the level of GCA. Indeed, substantial evidence has already demonstrated that an elevation in circulating bile acids levels seems to indicate improved energy homeostasis. For example, decreased circulating bile acids were observed in HFD-fed SD rats [58]. Particularly, HFD feeding was also found to lower circulating levels of CA, DCA [58], ω-muricholic acid (MCA), and α-MCA [59]. In contrast, this HFD-induced decrease in plasma bile acids could be improved in neurotensin-deficient mice [60]. Similarly, another study reported that compared with a high glycemic load diet, a diet low in glycemic load led to increases in circulating TLCA, TCA, and GCA, which may have beneficial effects on glucose homeostasis [61]. Naproxen, a COX inhibitor, combined with omega-3 polyunsaturated fatty acids were also found to significantly increase the plasma level of glycine-conjugated hyodeoxycholic acid, a secondary BA with a hypolipidemic effect [62]. Meanwhile, vertical sleeve gastrectomy (VSG) may also produce metabolically favorable alterations by increasing the level of circulating bile acid [39]. Moreover, an increase in circulating cholic acid was reported to result in decreases in endoplasmic reticulum stress with improved glucose homeostasis after ileal interposition surgery in UCD-T2DM rats [63]. Taken together, these studies support our findings that upregulation of circulating bile acids may be associated with beneficial metabolic effects in HFD-induced obese rodent models.

Admittedly, there also existed opposite findings about the relationship between energy dyshomeostasis and circulating bile acid levels. For instance, a previous study reported that serum bile acid concentrations in patients with type 2 diabetes were significantly higher than in healthy individuals [64]. Another study also showed that, compared with normal and anorexic subjects, the obese had higher levels of total plasma bile acids [65]. One possible reason for this discrepancy between studies might be that different metabolic disorders (e.g., HFD-induced obesity vs. type 2 diabetes) may result in different (even opposite) circulating bile acid profiles. Therefore, further research should be undertaken to more closely examine the associations between HACS-induced increases in circulating bile acids and its health benefits.

Notably, gut microbiota has been proven to play crucial roles in bile acid metabolism, thus fundamentally affecting circulating bile acid levels. Grau et al., reported that gut microbiota will significantly affect the metabolism and re-absorption of bile acids [66]. Gu et al., also demonstrated that there existed a close association between acarbose-dependent alterations in gut microbiota and serum BA composition [67]. Here, using Spearman correlation analysis, the obtained results also suggested the relative abundances of gut microbiota are important factors associated with the circulating levels of bile acids. Indeed, previous studies also reported similar findings in which there existed a close association between gut microbes and serum bile acids. For example, *Lactobacillus plantarum* isolated from food tended to alter glycol-conjugated BAs and produced free bile acids, in particularly DCA, CDCA, UDCA, and LCA [68]. Meanwhile, sophora flavescens Aiton ethyl acetate extract (SFE-H) treatment (at 200 mg/kg) was found to increase the abundance of *norank_f__Muribaculaceae* in mice with ulcerative colitis, with increases in bile acids (CA, UDCA and 12-KDCA) also being observed [69]. Therefore, the regulatory effects of HACS on the gut microbiota may be an important mechanism related to its influence on circulating bile acid profiles.

## 4. Materials and Methods

### 4.1. Materials and Reagents

The HACS was obtained from Xiangyu Biotech Co., Ltd. (Beijing, China), with amylose content being about 72%, and its molecular weight was in the range of 20,000 to 50,000. ICR mice (male), normal diet (4.60% fat), and high-fat diet (48.4% energy from fat (soy oil)) were purchased from Wushi Experimental Animals Co., Ltd. (Fuzhou, Fujian, China). The calorie density of the normal diet and high-fat diet were around 3.42 Kcal/g and 4.30 Kcal/g, respectively (Table S1). Anhydrous ethanol, methanol, and other reagents were all analytical purity grade or above.

### 4.2. Animal Experiments

Male ICR mice (2~3 weeks of age, 18~22 g) were kept under controlled environmental conditions (temperature 23 ± 1 °C, relative humidity 55 ± 5% and 12 h light/dark cycle). After 1 week of acclimation, the mice were randomly divided into 5 groups and subjected to the different treatment for 7 weeks: ND group (normal diet), HFD group (high-fat diet), INU group (HFD + 0.5 g/(kg·d) inulin, served as positive control), HACS_L group (HFD + 0.25 g/(kg·d) HACS) and HACS_H group (HFD + 0.5 g/(kg.d) HACS) (Figure S1). The HACS and inulin were administered by the intragastric (i.g.) route according to the method reported by Song et al. [70]. The intragastric administration was performed between 8:30 and 9:30 every morning. Body weight and food intake were measured every three days during the experiment. At the end of treatment, mice were fasted for 12 h, weighed and sacrificed by cervical dislocation. Blood was collected from eyeballs into a centrifuge tube and centrifuged at 3000 rpm to obtain the serum. The levels of triglyceride (TG), total cholesterol (TC), high density lipoprotein cholesterol (HDL-C), low density lipoprotein cholesterol (LDL-C), alanine aminotransferase (ALT), and aspartate aminotransferase (AST) were analyzed using an automatic biochemical analyzer (Hitachi High-Technologies Corporation, Tokyo, Japan); and the hepatic TG level was determined according to a previous study [71] using commercial kits (Applygen Technologies Inc., Beijing, China).

### 4.3. Hematoxylin-Eosin Staining

After sacrifice, liver, epididymal, and subcutaneous adipose tissues were dissected, weighed, and immediately fixed with 4% paraformaldehyde before being embedded in paraffin. The tissue depots were then sectioned into 5 μm thickness. After dewaxation, the slices were stained with hematoxylin and eosin (H&E). The images were recorded using an optical microscope at 400× magnification (Nikon, Tokyo, Japan), and the morphometric parameters were further analyzed using Image J software.

### 4.4. Oil Red O Staining

After sacrifice, liver, epididymal, and subcutaneous adipose tissue were dissected, weighed, and immediately fixed with 4% paraformaldehyde, then embedded in OCT compound and sectioned into 8 μm thickness. The slices were stained with Oil Red O for 5 min before being treated with hematoxylin dye for 10 s. The images were recorded using an optical microscope at 400× magnification (Nikon, Tokyo, Japan).

### 4.5. 16S rDNA Sequencing

Microbial DNA was extracted from the mice intestinal contents using E.Z.N.A.^®^ bacteria DNA Kit (Omega Bio-tek, Norcross, GA, USA) according to the manufacturer’s protocol. The V3-V4 hypervariable regions of the bacteria 16S rRNA gene were amplified with the primers listed in Table 2. The PCR products was extracted from a 2% agarose gel and further purified using the AxyPrep DNA Gel Extraction Kit (Axygen Biosciences, Union City, CA, USA) and quantified using QuantiFluorTM- ST (Promega, Casselton, ND, USA) according to the manufacturer’s protocol. Samples with bright strips between 400 and 450 bp were chosen for further analyses. The amplicon library was prepared with a Sample Preparation Kit (Illumina, San Diego, CA, USA) and sequenced on an Illumina Miseq platform according to standard protocols. Sequences with 97% similarity were defined as an operational taxonomic unit (OTU) and the representative sequence reads of various OTUs were hierarchically classified into different taxa. The obtained 16S data were uploaded to the NCBI database for public access (https://www.ncbi.nlm.nih.gov/sra/PRJNA826721 (accessed on 1 March 2022)).

### 4.6. Serum Bile Acids Measurement

For quantification of serum bile acids, 40 μL serum samples were spiked with 200 μL methanol in a 1.5 mL centrifuge tube and vortexed for 15 s and then incubated at −20 °C for 20 min. The mixture was then centrifuged at 14,000× *g* at 4 °C for 15 min. The supernatant was transferred to a new tube and dried at 60 °C in a vacuum concentrator for 45 min. The obtained residue was then dissolved in 100 μL methanol/water (1:1, *v*:*v*) and loaded into an automatic injector (at a 5 μL injection volume) of QTRAP 6500 LC-MS/MS System (AB SCIEX, Framingham, MA, USA) equipped with a Phenomenex C18 column for bile acids analysis. Water with 0.1% formic acid (phase A) and acetonitrile with 0.01% formic acid (phase B) was used as the phase at a flow rate of 300 μL/min at 35 °C. The gradient mobile phase was set as: 0–12 min, phase B from 25% to 40%; 12–14 min, phase B from 40% to 75%; 14–20 min, phase B from 75% to 100%; 20–22 min, phase B at 100%; 22–24 min, phase B from 100% to 25%; and 24–26 min, phase B at 25%. All data were collected in negative ion mode. MRM mode was used to detect the ion pair. The determination of bile acid concentration was performed by constructing a standard curve for absolute quantification.

### 4.7. Data and Statistical Analysis

The data were statistically analyzed using Graphpad Prism 8.0.1 (Graphpad Software Inc., La Jolla, CA, USA) and are expressed as mean ± variance (SD). Significant differences were determined using the Student’s *t*-test or ANOVA with post-hoc Tukey test. Differences were considered as significant at *p* < 0.05.

## 5. Conclusions

To sum up, HACS may profoundly influence the composition of circulating bile acids by regulating intestinal microbes, which may be associated with its regulatory effects on the physiological and biochemical indexes in obese mice with high-fat diets. The obtained results strongly suggested the underlying mechanisms of anti-obesity activity of HACS may involve its regulatory effects on gut microbiota and circulating bile acids, which may provide novel evidence to understand the metabolic benefits of HACS.

## Figures and Tables

**Table 1:** The circulating levels of bile acids in mice.

Bile Acid	ND (mmol/L)	HFD (mmol/L)	INU (mmol/L)	HACS-H (mmol/L)
γ-MCA	59.60 ± 13.27 ^a^	8.513± 0.2546 ^b^	15.46± 1.4539 ^b^	54.86± 0.9121 ^a^
α-MCA	4.228 ± 0.9557 ^a^	2.960 ± 0.01369 ^b^	3.135 ± 0.2584 ^b^	3.022 ± 0.1018 ^b^
7-KDCA	4.800 ± 1.288 ^a^	1.398 ± 0.1108 ^b^	2.199 ± 0.07913 ^b^	5.576 ± 0.7118 ^a^
67-DLCA	5.140 ± 1.088 ^b^	7.275 ± 0.8601 ^a^	6.800 ± 0.9369 ^a^	6.903 ± 0.6284 ^a^
HDCA	130.4 ± 16.29 ^a^	52.95 ± 6.372 ^c^	66.75 ± 9.071 ^bc^	76.65 ± 6.866 ^b^
UDCA	6.230 ± 1.179 ^a^	1.805 ± 0.4065 ^b^	2.471 ± 0.3706 ^b^	2.924 ± 0.2933 ^b^
DCA	25.01 ± 5.755 ^a^	12.10 ± 2.031 ^b^	16.47 ± 0.7915 ^b^	14.00 ± 1.031 ^b^
CDCA	4.095 ± 1.462 ^a^	1.158 ± 0.7083 ^b^	2.408 ± 0.1880 ^b^	2.473 ± 0.15331 ^b^
7-KLCA	2.974 ± 0.3200 ^a^	1.710 ± 0.1608 ^c^	2.279 ± 0.1656 ^b^	2.340 ± 0.1811 ^b^
TCA	44.80 ± 3.497 ^a^	25.79 ± 3.125 ^c^	41.96 ± 2.709 ^a^	36.48 ± 2.639 ^b^
TUDCA	10.54 ± 0.9519 ^a^	6.050 ± 0.4776 ^d^	7.284 ± 0.2137 ^c^	9.172 ± 0.2189 ^b^
TCDCA	55.44 ±3.551 ^a^	29.67 ± 2.020 ^c^	37.69 ± 1.694 ^b^	41.41 ± 0.6815 ^b^
GCA	0.5705 ±0.08908 ^ab^	0.7385 ± 0.2154 ^a^	0.5208 ± 0.01890 ^b^	0.5160 ± 0.01957 ^b^
β-MCA	9.215 ±0.4407 ^a^	6.465 ± 0.1040 ^c^	6.776 ± 0.2600 ^c^	7.549 ± 0.1657 ^b^
CA	49.61 ±1.447 ^a^	6.131 ± 4.338 ^c^	17.56 ± 0.3501 ^b^	20.63 ± 1.338 ^b^

Notes: ND, normal diet; HFD, high fat diet; HACS-L, high fat diet + 0.25 g/kg HACS; HACS-H, high fat diet + 0.5 g/kg HACS; and INU, high fat diet + 0.5 g/kg inulin. Data were expressed as the mean ± SD (*n* = 5 per group). Different superscripts represent significant differences (*p* < 0.05), and the same superscripts represent no significant differences (*p* ≥ 0.05).

**Table 2:** PCR amplification primers.

PCR Amplification Primers	Content
PCR amplification primer 1	338F (5′-ACTCCTACGGGAGGCAGCAG-3′)
PCR amplification primer 2	806R (5′-GGACTACHVGGGTWTCTAAT-3′)

## Data Availability

Data are contained within the article. The 16s ribosomal sequencing data can be accessed via the link: https://www.ncbi.nlm.nih.gov/sra/PRJNA826721 (accessed on 1 March 2022).

## Supplementary Materials

The following are available online at https://www.mdpi.com/article/10.3390/ijms23115905/s1.
