# Supplementary material for: High-Amylose Corn Starch Regulated Gut Microbiota and Serum Bile Acids in High-Fat Diet-Induced Obese Mice
Source: Int J Mol Sci. 2022 May 25;23(11):5905. doi: 10.3390/ijms23115905 (PMC9180756; doi:10.3390/ijms23115905)
Supplement: Supplementary file 1 [file ijms-23-05905-s001.zip › ijms-1647118-supplementary.pdf]

Supplementary Materials

Table S1. Nutritional composition of normal diet (ND) and high-fat diet (HFD).

composition	ND		HFD	
	percentage	Energy supply ratio	percentage	Energy supply ratio
fat (from soy oil)	4.60%	12.11%	18.40%	48.40%
protein	21.40%	22.47%	21.60%	32.00%
carbohydrates	55.90%	65.42%	43.10%	19.60%
fiber	4.00%		5.70%	
Ca	1.19%		1.70%	
P	0.87%		1.10%	

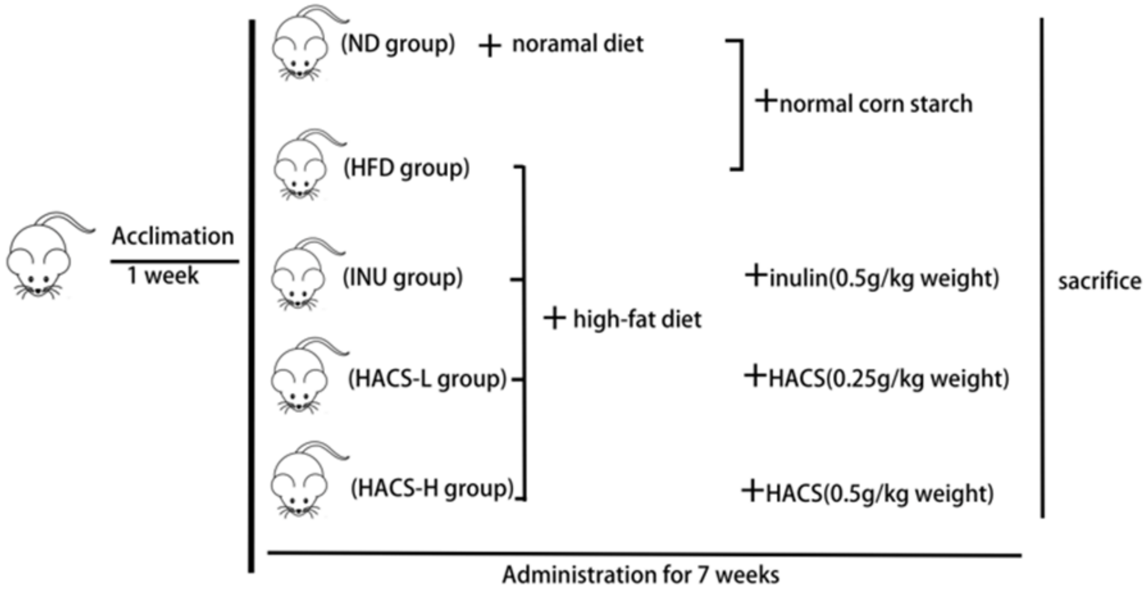

Figure S1. Administration of Mice for 7 Weeks. After 1 week of acclimation, the mice were divided into 5 groups randomly and subjected to the different treatment for 7 weeks. The HACS and inulin were administered by the intragastric (i.g.) route. The intragastric administration was performed between 8:30 and 9:30 every morning. At the end of treatment, mice were fasted for 12 hours, weighed and sacrificed by cervical dislocation.
